# Nanoparticle Albumin Bound Paclitaxel in the Treatment of Human Cancer: Nanodelivery Reaches Prime-Time?

**DOI:** 10.1155/2013/905091

**Published:** 2013-05-02

**Authors:** Iole Cucinotto, Lucia Fiorillo, Simona Gualtieri, Mariamena Arbitrio, Domenico Ciliberto, Nicoletta Staropoli, Anna Grimaldi, Amalia Luce, Pierfrancesco Tassone, Michele Caraglia, Pierosandro Tagliaferri

**Affiliations:** ^1^Medical Oncology Unit, Department of Experimental and Clinical Medicine, University “Magna Graecia” of Catanzaro and “Tommaso Campanella” Cancer Center, Campus Salvatore Venuta, Viale Europa, 88100 Catanzaro, Italy; ^2^Institute of Neurological Science (ISN-CNR), UOS of Pharmacology, Roccelletta di Borgia, 88021 Catanzaro, Italy; ^3^Department of Biochemistry, Biophysics and General Pathology, Second University of Naples, 80138 Naples, Italy

## Abstract

Nanoparticle albumin bound paclitaxel (nab-paclitaxel) represents the first nanotechnology-based drug in cancer treatment. We discuss the development of this innovative compound and report the recent changing-practice results in breast and pancreatic cancer. A ground-breaking finding is the demonstration that nab-paclitaxel can not only enhance the activity and reduce the toxicity of chromophore-diluted compound, but also exert activity in diseases considered refractory to taxane-based treatment. This is the first clinical demonstration of major activity of nanotechnologically modified drugs in the treatment of human neoplasms.

## 1. Introduction

Current development of cancer treatment mainly relies on three avenues:

- the identification of molecular targets for selective blockade of driver pathways in cancer cells or in tumour microenvironment,
- immunemodulatory approaches which might enhance the antitumor specific immune response,
- new delivery approaches in order to achieve higher bioavailability of anticancer agents.

The topic of the current review is the nanoparticle albumin bound paclitaxel (nab-paclitaxel) development, which has opened a novel scenario in cancer treatment by the enhancement of paclitaxel delivery by the use of nanotechnology.

## 2. Taxane (First) Revolution of Cancer Therapy

**Figure 1:**
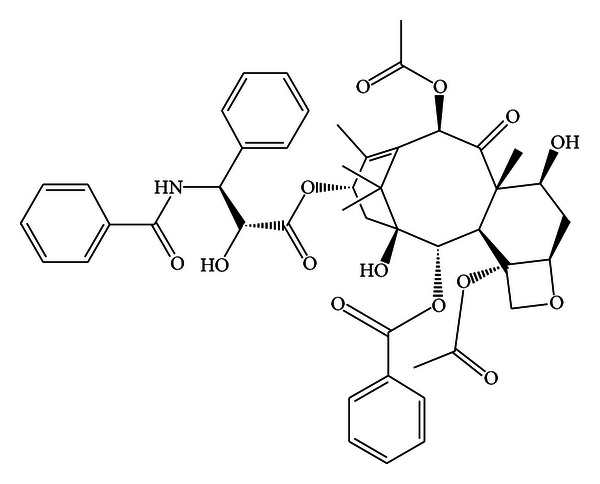
Structure of paclitaxel (5*β*,20-epoxy-1,2*α*,4,7*β*,13*α*-hex-ahydroxytan-11-en-9-one-4,10-diacetate2-benzoate-13-ester with (2*R.*3*S*)-*N-*benzoyl-3-phenyllioserine).

Taxanes are an important class of antitumor agents using solvent-based delivery vehicles. Paclitaxel (Bristol-Myers Squibb (New York, NY)) was identified in 1966, as an extract from *Taxus brevifolia*, obtained in a pure form in 1969 but its structure was published in 1971. Investigators faced several problems due to low concentration and structure complexities for low water solubility [1, 2] (Figure 1).

**Figure 2:**
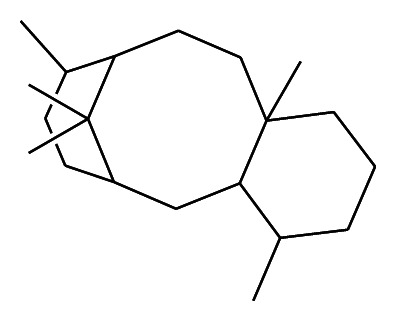
Taxane nucleus.

In fact, only in 1979 Susan Horwitz discovered that paclitaxel has a unique mechanism of action and interest which was additionally stimulated when impressive activity was demonstrated in NCI tumor screening [3]. Paclitaxel is a diterpenoid pseudoalkaloid with formula C_47_H_51_NO_14_ (*MW* = 853 Da) whose activity was demonstrated in different preclinical models. For antitumor activity the presence of the entire taxane molecule is required (Figure 2) for the inactivity of the ester and the tetraol formed by a low temperature cleavage of paclitaxel [4].

Although the development of paclitaxel was hampered by limited availability of its primary source and the difficulties inherent to large-scale isolation, extraction, and its poor aqueous solubility, interest was maintained after characterization of its novel mechanism of cytotoxic action. In order to afford new preclinical and clinical studies, it was necessary to find new and more abundant and renewable resources. These studies led to the development of docetaxel (Taxotere), a semisynthetic taxane analogue extracted from *Taxus baccata*, a European yew. Docetaxel differs from paclitaxel in two positions in its chemical structure and this small alteration makes it more watersoluble. Taxanes disrupt microtubule dynamics by stabilizing the microtubule against depolymerization, enhancing their polymerization, promoting the nucleation and elongation phases of the polymerization reaction, and reducing the critical tubulin subunit concentration required for microtubule assembly. Moreover they alter the tubulin dissociation rate at both ends of the microtubule. This leads to reduced dynamic instability, whereas the association rate is not affected. After the treatment with taxanes, the microtubules are highly stable and resistant to depolymerization by cold, calcium ions, dilution, and other antimicrotubule agents. The final result is the impairment of dynamics of microtubule depolymerization, which is a critical event in the mitotic process [5].

Paclitaxel is active against primary epithelial ovarian carcinoma, breast cancer, colon, non-small-cell lung cancer, and AIDS-related Kaposi's sarcoma in preclinical models [3, 6, 7] and is presently of common use in the treatment of several important malignancies as lung cancer, breast cancer, Kaposi's sarcoma, squamous cell carcinoma of the head and neck, gastric cancer, esophageal cancer, bladder cancer, and other carcinomas. Despite being clinically very active, paclitaxel and docetaxel are associated with many serious sideeffects which often preclude the prolonged use in patients. A number of these side effects have been associated with the vehicles used for the formulation: the cremophor EL (CrEL-polyethoxylated castor oil) [8] for paclitaxel and polysorbate 80 (Tween 80) for docetaxel, respectively, that altered also their pharmacokinetic profiles; CrEL is considered to be responsible for the hypersensitivity reactions seen in patients during paclitaxel therapy. *In vitro*, CrEL caused axonal swelling, demyelination, and axonal degeneration, and, thus, it may also contribute to the development of neuropathy in patients receiving paclitaxel. The use of CrEL requires premedication with antihistamines and corticosteroids to prevent hypersensitivity reactions and, despite these premedications, approximately 40% of all patients will have minor reactions (e.g., flushing and rash) and 3% will have life threatening reactions. CrEL also causes leaching of the plasticizers from polyvinyl chloride (PVC) bags and infusions sets; thus paclitaxel must be infused via the use of special non-PVC infusion systems and in-line filtration. Another effect induced by CrEL is the alteration of lipoprotein pattern and the consequent hyperlipidemia. Moreover, CrEL and polysorbate 80 interfere with efficacy by limiting tumor penetration through the formation of large polar micelles, which for CrEL-paclitaxel can lead to nonlinear pharmacokinetics and decreased unbound drug fraction [9].

To overcome the ideal dosage form and bypass all the present limitations, novel “carrier delivery systems,” including liposomes, micelles, and particulate drug delivery systems, were formulated as common practice for novel drugs like microRNAs [10–15].

Some of them have already reached the clinical practice like liposomal doxorubicin or liposomal amphotericin B. Another example of nanotechnology applied to drug delivery is the preclinical development of stealth liposomes encapsulating zoledronic acid (LipoZOL) to reduce binding of ZOL to bone and increase its bioavailability in extraskeletal tumor sites [16]. Natural human protein based carrier can also be used to manufacture nanocarriers for drug delivery: this is the example of the paclitaxel albumin bound by which it is possible to selectively deliver larger amounts of drug to tumors, reducing the toxicities related to solvent-based formulations. Albumin is a natural carrier of hydrophobic endogenous molecules (such as vitamins, hormones, and other plasma constituents), in a noncovalent and reversible binding and allows for transport in the body and release at the cell surface [17].

Abraxane (nab-paclitaxel; ABI 007 or Abraxane; Celgene Inc, Odenton, MD,USA) was the first to receive FDA approval in 2005, for the treatment of breast cancer in patients who reported progressive disease after chemotherapy for metastatic cancer or relapse within 6 months of adjuvant chemotherapy.

Nab-paclitaxel is a colloidal suspension of 130 nanometer particles, solvent-free, homogenized with human serum albumin (3%-4%), by which it is possible to infuse higher doses of drug than the standard dose used in paclitaxel therapy, with fewer side effects, with less infusion time (30 minutes) and without premedication. The new formulation allows the delivery of paclitaxel to tumors with a 4.5-fold increase in its transport, coupled with albumin receptors, across endothelial cells [18] with an enhanced intracellular antitumor paclitaxel delivery and activity [19]. In the mechanism of drug delivery an albumin receptor (gp60) on endothelial cells seems to be involved which transports paclitaxel into the extravascular space with subsequent invagination of the cell membrane to form caveolae, transcytotic vesicles, and also tumor accumulation of nanoparticle bound to SPARC (secreted protein, acidic and rich in cysteine), which is overexpressed in many solid tumors, including bladder, prostate, and pancreas cancers [20]. Its intravenous infusion is more manageable and safe because it is performed by standard plastic intravenous infusion bags and can also be reconstituted in a much smaller volume of normal saline compared to paclitaxel. Preclinical studies have demonstrated that nab-paclitaxel achieved higher intratumor concentrations compared to CrEL-paclitaxel with a better bioavailability and showed an improved efficacy and therapeutic index in multiple animal models [21]. Other new technologies recently used to deliver paclitaxel have led to the development of innovative formulations such as Nanoxel and liposomal and polymeric paclitaxel.

Nanoxel-PM is efficacious and less toxic than free docetaxel formulation and was evaluated in comparison with Taxotere in preclinical studies. Nanoxel-PM can reduce sideeffects of hypersensitivity reactions and fluid retention while retaining antitumor efficacy in cancer patients [22].

Further studies led to the development of new formulations of liposomal paclitaxel. The special composition of the liposomal membrane which contains high doses of paclitaxel could reduce the aggregation giving the molecule higher stability and confers an increase of efficacy in animal models as in human tumors [23].

An hydrotropic polymer micelle system has also been developed for delivery of poorly water-soluble drugs as paclitaxel. This polymer showed not only higher loading capacity but also enhanced physical stability in aqueous media and provides an alternative approach for formulation of poorly soluble drugs [24, 25].

## 3. Nab-Paclitaxel in Breast Cancer Treatment

Breast cancer (BC) is the most common cancer in female patients and follows lung cancer as the most common cause of female cancer death. While only 5–7% of BC patients present metastatic disease (mBC) at diagnosis and more than 30% presenting localized disease will eventually recur, 5 year survival of advanced disease is less than 20% [33]. Current treatment of advanced breast cancer is mainly aimed to ameliorate quality of life and prolong survival. Treatment choice is not an easy task in terms of drug selection and combination. Chemotherapy plays an essential role for the treatment of mBC. Among anticancer drugs, taxanes are considered the most effective, while their use involves long infusion time, neurotoxicity, and high risk of hypersensitivity reactions [8, 34, 35]. These latter effects are due to allergic reactions induced by the use of solubilizing agents (as chromophores) and today are less common due to the use in the clinical practice of corticosteroids and antihistamines [36]. In order to overcome these important limitations, a major interest is devoted to novel drugs as nab-paclitaxel, eribulin, ixabepilone, PARP inhibitors, and new HER 2 inhibitors as lapatinib, pertuzumab, TDM1, and neratinib [37–43].

Following phase I studies, by Ibrahim et al. in 2002 [19] and by Teng et al in 2004 [44], which led to MTD identification at 300 mg/m^2^ in the three weekly schedule with neurotoxicity as dose limiting toxicity, Nyman et al. in 2005 [45] identify in the weekly schedule the MTD at 100 mg/sqm for highly pretreated patients and 150 mg/m^2^ for nonhighly pretreated patients with grade 4 neutropenia and grade 3 neuropathy as DLT with earlier onset at higher dosages. The pivotal phase 3 study was published in 2005 where Gradishar et al. [30] compared nab-paclitaxel (260 mg/m^2^) at three week schedule with CrEL-paclitaxel 175 mg/m^2^ also at three week schedule. The study clearly demonstrated a survival advantage for nab-paclitaxel with an improved toxicity profile.

In 2009 a phase II randomized study [26] compared three week docetaxel 100 mg/m^2^ with three week nab-paclitaxel 300 mg/m^2^, weekly nab-paclitaxel 100 mg/sqm and weekly nab-paclitaxel 150 mg/sqm. The 150 nab-paclitaxel weekly schedule provided the best PFS (>5 months) which resulted to be statistically significant. An update of this study published by Gradishar et al. in 2012 demonstrated a median overall survival (OS) of 33.8 months which statistically overcame the other treatment arms.

All together these data demonstrated that nab-paclitaxel is superior to CrEL-paclitaxel in the three week schedule and that nab-paclitaxel at weekly 150 schedule provides an impressive long term survival [27]. Recently, nab-paclitaxel was administered in combination with biological agents in the treatment of mBC. In detail, a safety analysis of the first ten enrolled patients treated for at least one cycle of the initial doses of nab-paclitaxel (125 mg/m^2^ i.v. on days 1, 8, and 15 every 28 days) in combination with lapatinib (1,250 mg orally once daily on a continuous basis) in a 4-week cycle for a planned minimum of six cycles was performed. However, during the ongoing safety review of the first five patients, Grade 3 toxicities were observed in all five patients (four with neutropenia and one with neutropenic fever and diarrhea) and the decision was made to reduce the dose of both study drugs. All subsequent patients (*n* = 55) received nab-paclitaxel (100 mg/m^2^ i.v. on days 1, 8, and 15 every 28 days) in combination with lapatinib (1,000 mg orally once daily on a continuous basis) in a 4-week cycle for a minimum of six cycles. RR was 53% with the majority of patient responses demonstrating a partial response (PR) (47%). Four (7%) patient responses demonstrated a complete response (CR), and ten (17%) demonstrated a stable disease. The progression-free survival (PFS) and time to progression (TTP) were 39.7 weeks (95% CI 34.1–63.9) and 41 weeks (95% CI 39.1–64.6), respectively. Lapatinib 1,000 mg with nab-paclitaxel 100 mg/m^2^ i.v. is feasible with manageable and predictable toxicity and an RR of 53% comparing favorably with other HER2-based combinations in this setting [50].

Two important points under investigation are the comparison of weekly nab-paclitaxel with CrEL-paclitaxel both at weekly schedules and the potential advantage of combination with bevacizumab. Finally nab-paclitaxel has shown some activity also in CrEL-paclitaxel heavily pretreated and resistant patients [28] (Table 1).

## 4. Nab-Paclitaxel in Pancreatic Cancer Treatment

Pancreatic cancer (PC) is at present a big cancer killer, with an expected survival of 6 months in advanced stage PC (aPC). Till a recent report demonstrating good activity of oxaliplatin, irinotecan, and fluorofolate (FOLFIRINOX combination), gemcitabine is still the mainstay treatment. In a recent meta-analysis, Ciliberto et al. [51] described a statistically superiority in terms of survival and response rate for gemcitabine-based combination compared to gemcitabine alone. Moreover, this advantage was marginal and at the cost of an increased toxicity. The authors concluded that in the era of targeted therapy new approaches were possible only in presence of solid preclinical findings.

A report by von Hoff et al. [31] demonstrated in a phase I/II study an interesting activity of gemcitabine/nab-paclitaxel combination at gemcitabine 1000 mg/m^2^ and nab-paclitaxel at 125 mg/m^2^ doses weekly for three doses in a 4 week schedule. A 48% response rate was achieved at MTD. The authors additionally demonstrated that SPARC-expressing tumors appeared more sensitive to the drug combination.

An interesting finding from a preclinical study reported that nab-paclitaxel demonstrated the capacity of increasing the gemcitabine bioavailability inside the tumors. These findings led to the design of a phase III study where gemcitabine/nab-paclitaxel was compared to gemcitabine alone showing an advantage in OS, PFS, and RR. This study, presented to ASCO GI 2013 (American Society of Clinical Oncology, Gastrointestinal Cancer Symposium) by von Hoff, is clearly a changing practice study and the gemcitabine/nab-paclitaxel, which led to an almost two month longer OS should be now compared to FOLFIRINOX combination (Table 2). The biological bases of the synergistic interaction between nab-paclitaxel and gemcitabine have recently been elucidated by an *in vivo* study in animal models. In detail, the combination treatment was administered to KPC mice that develop advanced and metastatic pancreas ductal adenocarcinoma. The authors have demonstrated an increase of intratumoral gemcitabine levels attributable to a marked decrease in the primary gemcitabine metabolizing enzyme, cytidine deaminase. Correspondingly, paclitaxel reduced the levels of cytidine deaminase protein in cultured cells through reactive oxygen species-mediated degradation, resulting in the increased stabilization of gemcitabine. These findings support the concept that suboptimal intratumoral concentrations of gemcitabine represent a crucial mechanism of therapeutic resistance in PC [52]. This study provides mechanistic insight into the clinical cooperation observed between gemcitabine and nab-paclitaxel in the treatment of pancreatic cancer.

## 5. Other Areas of Nab-Paclitaxel Development

Melanoma represents 5% and 4% of all cancers in males in females, respectively. However, the rates of incidence of melanoma are steadily increasing in the USA as in most parts of Europe [53]. The survival rates of melanoma become worse with advancing stage. Therefore, early diagnosis in addition to surgical treatment before its spread is the most effective treatment.

Melanomas are a heterogeneous group of tumors characterized by specific genetic alterations, including mutations in kinase, such as BRAF or c-kit. Dacarbazine is commonly used as a treatment for metastatic melanoma and has been for long time the standard of care for this disease. Recently, new approaches have completely changed the diagnosis and treatment of melanoma. New medications like vemurafenib have been developed for the systemic therapy of advanced melanomas in subpopulations identified by BRAF mutation tests. Taxanes have been reported to have some limited activity in malignant melanoma [54–58], due to the high toxicity attributed to their waterinsolubility. In a phase II clinical trial Hersh at al. in 2010 [46] demonstrated that nab-paclitaxel has activity not only in chemotherapy-naïve patients with metastatic melanoma administered at a dose of 150 mg/m^2^ but also in previously treated patients administered at a dose of 100 mg/m^2^ for 3 of 4 weeks. In this study, PFS and OS were longer than the previous results reported with conventional standard of care. In previously treated and chemotherapy-naïve patients, PFS was 4.5 months and 3.5 months, respectively, and similarly OS was 9.6 months and 12.1 months (in respect to 1.6 months of PFS reported in the literature for treatment with dacarbazine and temozolomide). In another phase II clinical trial, Kottschade et al. in 2011 [59] demonstrated that in patients with metastatic melanoma the combination of nab-paclitaxel 100 mg/m^2^ and carboplatin AUC2 administered in days 1, 8, and 15 every 28 days is moderately tolerated for the occurrence of adverse effects that were fatigue, myelodepression, and gastrointestinal toxicity. This study confirms that the efficacy and toxicity of nab-paclitaxel are similar to those of paclitaxel when combined with carboplatin for the treatment of patients with metastatic melanoma. Even if such regimens have not been formally compared in a randomized study, we can say that nab-paclitaxel is a good alternative for patients who cannot tolerate conventional therapy with paclitaxel. Last November at the Society of Melanoma Research a preliminary analysis of a Phase III study by Hersh was presented which shows benefit in terms of PFS in favor of nab-paclitaxel compared to dacarbazine (4.8 versus 2.5 months); the same trend was observed in the interim analysis that shows a trend for better OS (12.8 versus 10.7 months) (Table 3). Recently, nab-paclitaxel was efficiently combined with temozolomide and oblimersen in the treatment of melanoma patients. In detail, in a phase I trial, chemotherapy-naïve patients with metastatic melanoma and normal LDH levels were enrolled in 3 cohorts. The treatment regimen consisted of 56-day cycles of oblimersen (7 mg/kg/day continuous i.v. infusion on days 1–7 and 22–28 in cohort 1 and 2; 900 mg fixed dose, twice weekly in weeks 1-2, 4-5 for cohort 3), temozolomide (75 mg/m^2^, days 1–42), and nab-paclitaxel (175 mg/m^2^ in cohort 1 and 3, 260 mg/m^2^ in cohort 2 on days 7 and 28). The RR in the 32 treated patients was 40.6% (2 CR and 11 PR) and 11 patients had stable disease, for a disease control rate of 75%. Haematological, renal, and neurologic toxicity never exceeded grade 3 demonstrating a good tolerability of the schedule [60].

Lung cancer (LC) is the first cause of cancer death all over the world, with a 5 year survival of 5% for metastatic disease. Treatment selection is based on different factors like the performance status, comorbidities, histology, and, in the last years, the molecular mutational profile, which is now mandatory to assess before deciding treatment. The most common chemotherapy approach is a platinum based doublet which is commonly combined with gemcitabine, vinorelbine, or pemetrexed [61] in Europe, while in the USA the most common combination is carboplatin paclitaxel doublet (RR 15–32%); this combination is effective and relatively well tolerated in the elderly [62–65]. Bevacizumab addition to this combination led to improved survival [66]. Socinski et al. reported in 2012 a phase III trial enrolling 1052 IIIb aNSCLC (advanced non-small-cell lung cancer) patients in the first line of treatment which compared weekly nab-paclitaxel 100 mg/m^2^ and carboplatin AUC6 every three weeks with carboplatin AUC6 and CrEL-paclitaxel 200 mg/m^2^ every three weeks [49]. The nab-paclitaxel/carboplatin combination was more active in terms of RR with a trend in PFS and OS improvement and was also better tolerated (Table 4).

## 6. Conclusions and Future Developments

Nab-paclitaxel has produced a paradigm change in the treatment of tumors like breast cancer, pancreatic cancer, and melanoma and a large use in these important diseases can be predicted. Also in lung cancer, nab-paclitaxel has produced a good safety profile and increase in RR.

We think that nab-paclitaxel has opened a new way to human cancer treatment and indeed reached the prime-time.

## Figures and Tables

**Table:** (a) Phase II

	Arms		Pts	RR (%) INV. RAD. *P* = .047	RR (%) IND. RAD. *P* = .047	PFS (%) INV. RAD. *P* = .047	PFS (%) IND. RAD. *P* = .047	OS (months) *P* = .47
Gradishar et al., 2009 [26] Gradishar et al., 2012 [27] Update OS (first line)	Nab-paclitaxel	300 mg/m^2^ q3w 150 mg/m^2^ qw 100 mg/m^2^ qw	76 74 76	46 74 63	37 49 45	10.9 14.6 7.5	11 12.9 12.8	27.7 33.8 22.2
	Docetaxel 100 mg/m^2^ q3w		74	39	35	7.8	7.5	26.6

	Arms		Pts	ORR (%)		Median PFS (months)		OS (months)
				*P* = .73		*P* = ND		*P* = .71

Blum et al., 2007 [28] (following lines)	Nab-paclitaxel 125 mg/m^2^ qw		75	16		3.5		9.1
	Nab-paclitaxel 100 mg/m^2^ qw		106	14		3.0		9.2

	Arm		Pts	RR I line (%) *P* = ND	RR > I line (%) *P* = ND	ORR (%) *P* = ND	Median TTP (weeks) *P* = ND	Median survival (weeks) *P* = ND

Ibrahim et al., 2002 [19] (first and following lines)	Nab-paclitaxel 300 mg/m^2^ q3w		63	64	21	48	26.6	63.6

	Arms		Pts	Median PFS (months) *P* = ND	PFS at 6 months (%) *P* = ND	MDR (months) *P* = ND	Median OS (months) *P* = ND	OS at 6 months (%) *P* = ND

Roy et al., 2009 [29] (first line)	Nab*-*paclitaxel 125 mg/sqm Gemcitabine 1000 mg/sqm days 1 and 8		50	7.9	60	6.9	Not reached	92

**Table:** (b) Phase III

					AEs (%) *P* = .001	
	Arms	Pts	RR (%) *P* = .001	TTP weeks *P* = .006	Grade IV neutropenia	Grade III sensory neuropathy
Gradishar et al., 2005 [30] (first line)	Nab-paclitaxel 260 mg/sqm	229	33	23.0	9	10
	Paclitaxel 175 mg/sqm	225	19	16.9	22	2

*P*: *P* value; nd: not done; AEs: adverse events; inv. rad.: investigator radiologist; ind. rad.: independent radiologist; ORR: overall response rate; RR: response rate; TTP: time to progression; PFS: progression-free survival; OS: overall survival; MDR: median duration of response.

**Table:** (a) Phase I/II

	Arms		Pts	MTD	RR (%) *P* = ND	Median OS (months) *P* = ND	1 year survival (%) *P* = ND
von Hoff et al., 2011 [31] (First line)	Gemcitabine1000 mg/sqmNab-paclitaxel	100 mg/m^2^ q3w 125 mg/m^2^ q3w 150 mg/m^2^ q3w	20 44 3	**X**	48	12.2	48

**Table:** (b) Phase III

	Arms	Pts	ORR (%)	Median TTP (MO)	PFS		OS			AEs (%) *P* = .001		
					Median (MO)	1 yr (%)	Median (MO)	1 yr (%)	2 yr (%)	Grade ≥ III neutropenia	Fatigue	Neuropathy
			*P* = <.001	*P* = <.001	*P* = <.001	*P* = .031	*P* = <.001	*P* = <.001	*P* = .02			
Von Hoff et al., 2011 [32] (first line)	Nab-paclitaxel 125 mg/m^2^ qw followed Gemcitabine 1000 mg/sqm qw	431	99	5.1	5.5	16	8.5	35	9	38	17	17
	Gemcitabine 1000 mg/sqm qw	430	31	3.6	3.7	9	6.7	22	4	27	7	1

*P*: *P* value; nd: not done; AEs: adverse events; MTD: maximum tolerated dose; ORR: overall responce rate; RR: response rate; TTP: time to progression; PFS: progression-free survival; OS: overall survival; MDR: median duration of response.

**Table:** (a) Phase II

	Arms		Pts	RR (%) P = .05	PFS		OS	
					Median (MO) P = ND	At 6 (%) P = ND	Median (MO) P = ND	1 year (%) P = ND
Hersh et al., 2010 [46] (first* and following** line)	Nab-paclitaxel	*150 mg/m^2^ q3w **100 mg/m^2^ q3w	37 37	21.6 2.7	4.5 3.5	34 27	9.6 12.1	41 49

	Arms		Pts	RR (%) P = .10	Median PFS (MO) P = ND		Median OS (MO) P = ND	

Kottschade et al., 2011 [47] (first* and following** line)	Nab-paclitaxel	*100 mg/m^2^ q3w Carboplatin AUC2	41	25.6	4.3		11.1	
		**100 mg/m^2^ q3w Carboplatin AUC2	35	8.8	4.2		10.9	

**Table:** (b) Phase III

	Arms	Pts	ORR (%)	PFS				OS				AEs grade ≥ III (%) *P* = .001			
				Median (MO)	BRAF status			Median (MO)	BRAF status			Neutropenia	Leukopenia	Fatigue	Neuropathy
					WT (MO)	V600Em (MO)	Uk (MO)		WT (MO)	V600Em (MO)	Uk (MO)				
			P = .239	P = .044	P = .088	P = .656	P = .066	*P* = <.001	P = .33	P = .132	P = .381				
Hersh et al., 2010 [48] (first line)	Nab-paclitaxel 150 mg/m^2^ qw	264	15	4.8	5.4	5.3	3.7	12.8	12.7	16.9	11.1	20	12	8	25
	Dacarbazine 1000 mg/sqm q3w	265	11	2.5	2.5	3.5	2.2	10.7	11.1	11.2	9.9	10	7	2	0

P: *P* value; nd: not done; AEs: adverse events; WT: wild type; V600Em: with mutation of V600E; Uk: unknown BRAF mutation; ORR: overall responce rate; RR: response rate; PFS: progression-free survival; OS: overall survival.

**Table 4:** Randomized phase III trials with nab-paclitaxel in aNSCLC.

	Arms	Pts	ORR			Median PFS (MO) *P* = <.214	Median OS (MO) *P* = <.271	AEs grade III*-IV** (%) *P* = <.001			
			Median (%) *P* = .005	SQ (%) *P*= <.001	NSQ (%) *P* = <.80			Neutropenia	Thrombocytopenia	Fatigue	Anemia
Socinski et al., 2012 [49] (first line)	Nab-paclitaxel 100 mg/m^2^	521	33	41	26	6.3	12.1	33*	13*	4*	22*
	Carboplatin AUC6 q3w							14**	5**	<1**	5**
	Paclitaxel 200 mg/m^2^	531	25	24	25	5.8	11.1	32*	7*	6*	6*
	Carboplatin AUC6 q3w							26**	2**	<1**	<1**

*P*: *P* value; nd: not done; sq: squamous histology of NSCLC; nsq: non squamous histology of NSCLC; AEs: adverse events; ORR: overall responce rate; RR: response rate; PFS: progression-free survival; OS: overall survival.
